# The pre-Columbian introduction and dispersal of Algarrobo (*Prosopis*, Section Algarobia) in the Atacama Desert of northern Chile

**DOI:** 10.1371/journal.pone.0181759

**Published:** 2017-07-24

**Authors:** Virginia B. McRostie, Eugenia M. Gayo, Calogero M. Santoro, Ricardo De Pol-Holz, Claudio Latorre

**Affiliations:** 1 Departamento de Antropología, Facultad de Ciencias Sociales, Pontificia Universidad Católica de Chile, Santiago, Chile; 2 Ecología & Centro UC Desierto de Atacama, Facultad de Ciencias Biológicas, Pontificia Universidad Católica de Chile, Santiago, Chile; 3 Instituto de Ecología y Biodiversidad, Santiago, Chile; 4 Departamento de Oceanografía, Universidad de Concepción, Concepción, Chile; 5 Center for Climate and Resilience Research (CR)2, Santiago, Chile; 6 Instituto de Alta Investigación, Laboratorio de Arqueología y Paleoambiente, Universidad de Tarapacá, Arica, Chile; 7 GAIA-Antartica, Universidad de Magallanes, Punta Arenas, Chile; University of Arkansas, UNITED STATES

## Abstract

Archaeological and palaeoecological studies throughout the Americas have documented widespread landscape and environmental transformation during the pre-Columbian era. The highly dynamic Formative (or Neolithic) period in northern Chile (ca. 3700–1550 yr BP) brought about the local establishment of agriculture, introduction of new crops (maize, quinoa, manioc, beans, etc.) along with a major population increase, new emergent villages and technological innovations. Even trees such as the Algarrobos (*Prosopis* section Algarobia) may have been part of this transformation. Here, we provide evidence that these species were not native to the Atacama Desert of Chile (18–27°S), appearing only in the late Holocene and most likely due to human actions. We assembled a database composed of 41 taxon specific AMS radiocarbon dates from archaeobotanical and palaeoecological records (rodent middens, leaf litter deposits), as well an extensive bibliographical review comprising archaeobotanical, paleoecological, phylogenetic and taxonomic data to evaluate the chronology of introduction and dispersal of these trees. Although Algarrobos could have appeared as early as 4200 yr BP in northernmost Chile, they only became common throughout the Atacama over a thousand years later, during and after the Formative period. Cultural and natural factors likely contributed to its spread and consolidation as a major silvicultural resource.

## Introduction

The impact of humans on the surface of the Earth over recent centuries has been so significant that there are concrete initiatives to formally define this recent timeframe as a new geological epoch or even era, aptly termed the “Anthropocene” [1–3]. Despite some consensus regarding its causes, the academic community remains divided on when this epoch began. Most authors consider a late onset by the Industrial Revolution [1, 2] and others after the Great Acceleration by the mid-20th century [4]. In contrast, some argue for a much older onset, first by hunter-gatherers followed by an intensification after the Formative period (the regional Neolithic epoch dated at 3700–1550 yr BP) when food production systems (e.g. husbandry and agriculture) rose in importance along with more permanent settlements [5–9].

We examined available evidence to determine whether Algarrobo trees belonging to *Prosopis*, Algarobia section (from here on we will use the term Algarrobo to include all the described species of this section) could have been introduced by humans from the Chaco Region (in modern Paraguay, Bolivia and Argentina) into the Atacama Desert during the late Holocene. This hypothesis challenges the current idea that this iconic tree was native in Chile, an assertion based on the presence and visibility of these resources from the Formative until the present [10–16]. Their importance for pre-Columbian groups has been emphasized by calling them “*the people of Chañar and Algarrobo*” [17]. These species, however, were largely absent before the Formative period [18–23]. A rapid spread from a single or several introductions would have transformed the desert landscape significantly. Such a spread could have been further facilitated by natural and/or cultural vectors in a context of increasing moisture availability [16, 24–26] along with major cultural transformations [27, 28].

### Taxonomy, distribution and biogeography of *Prosopis* L

The genus *Prosopis* (Fabaceae-Mimosoidea) is amphitropical and found throughout the arid and semiarid regions of the world. Around 44 species have been described, most of them native to the Americas with one from tropical Africa and three from Southeast Asia [29, 30]. Over 80% of these species occur in South America, grouped into three sections: Strombocarpa (eight species), Algarobia (30 species) and the monospecific Monilocarpa section (*Prosopis argentina*) in Argentina [29].

The disjunct distribution of *Prosopis* in the Americas has led to contrasting hypotheses regarding its evolutionary history. Most scholars agree that the main centre of diversity and polymorphism is the Argentine–Paraguayan–Chilean region. A secondary diversity centre has also been proposed in the Texan–Mexican region [29, 31–36]. The paleotropical hypothesis postulates that *Prosopis* spread from Africa to North America during the Paleogene, and then subsequently dispersed into South America [37]. In contrast, Simpson [36] proposed that ancestral *Prosopis* species crossed from Africa to South America and propagated into North America before the radiation of major known lineages.

Under this vicariant scenario, *Prosopis* experienced subsequent convergent evolution. Pasiecznik et al. [33], suggests that the *P*. *pallida/P*. *juliflora* complex occupies the middle ground between the Texan–Mexican and Argentinean–Paraguayan–Chilean centres; these dispersed to North America via the Andes from Argentina to Bolivia and from there into the hyperarid coast of Chile and southern Peru, before spreading northwards. In contrast, Bessega et al. [38], proposed ancestral areas for the American species in western USA, Mexico, Peru, Ecuador as well as central-northern Argentina, with further lineage fragmentations brought about by successive vicariance events.

**Fig 1:**
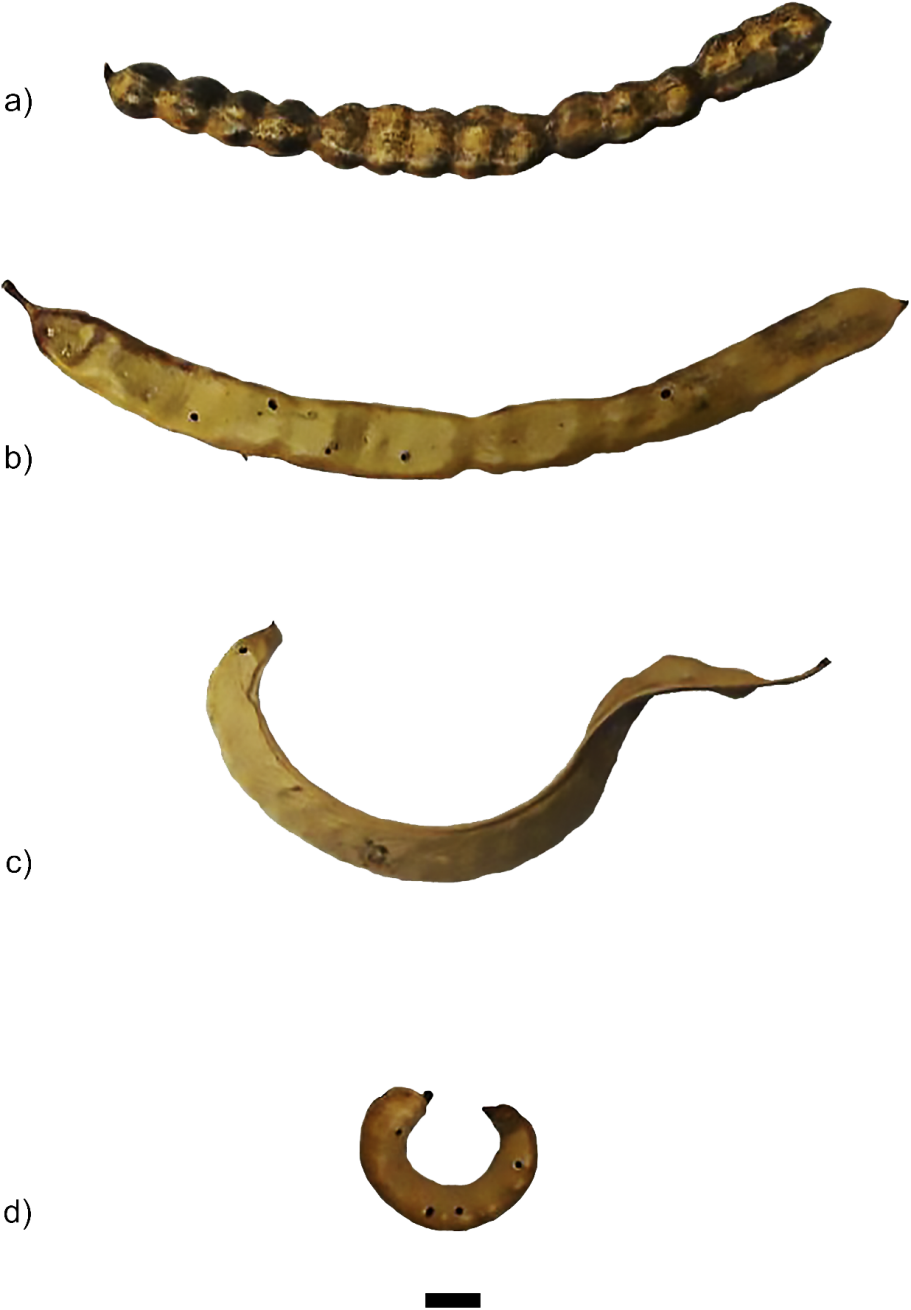
*Prosopis* sections seed pods collected by the authors in northern Chile. Scale bar represents 1 cm. a) cf. *Prosopis alba*; b) cf. *Prosopis flexuosa*; c) cf. *Prosopis chilensis*; d) *Prosopis tamarugo*.

The American main sections differ in morphological traits and phytogeographic history [30, 31, 39, 40] (Fig 1). For instance, the Strombocarpa section, divided into Strombocarpae and Cavenicarpae series can produce wood, shade, fuel and ‘screwbeans’ or “tornillos” seed pods that provide good fodder, but are not edible to humans. In contrast, the diverse Algarobia section includes valuable trees with a wide variety of uses such as shade, fuel, food and forage for wildlife, livestock and human consumption [29, 33, 41]. Hence, multiple social and economic benefits of the Algarobia section have motivated their deliberate introduction into different countries [33, 42, 43]. Burkart [29] divided this section into six series: Sericanthae, Ruscifoliae, Humiles, Denudantes, Pallidae and Chilenses. Their taxonomy, however, is confounded by elevated intraspecies phenotypic variation, low genetic differentiation, and frequent hybridization [33, 38, 40, 44]. Molecular clocks show that both sections diverged during the Oligocene, with a more recent event between the extant series during the Miocene. The diversification of Algarobia into the Ruscifoliae, Chilenses and Pallidae series likely occurred during the late Pliocene [32].

**Fig 2:**
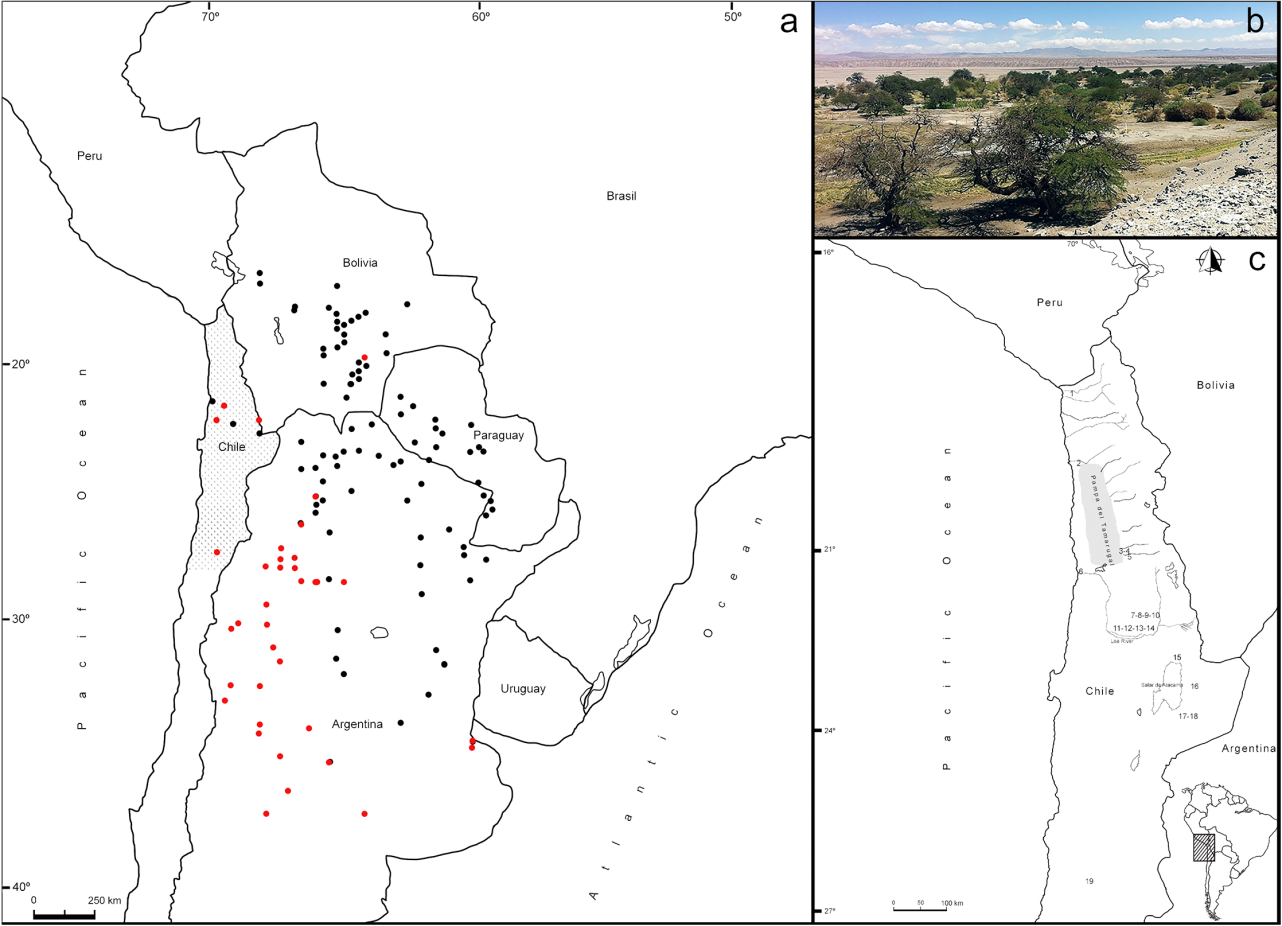
(A). The modern distribution of *Prosopis alba* (black dots) and *P*. *flexuosa* (red dots) section Algarobia in central South America (GBIF Secretariat: GBIF Backbone Taxonomy. doi:10.15468/39omei. Accessed via http://www.gbif.org/species/5358452 on 2017-04-10; http://www.gbif.org/species/5358528 on 2017-04-10). Note the widespread distribution in NW Argentina, eastern Paraguay and southern Bolivia compared to the distribution in northern Chile. (B) Algarrobos growing in an oasis of the Atacama Desert (C) Archaeological and palaeoecological sites dated in this study, see also S3 Table (1. Lluta 13; 2. Tiliviche 1B; 3. Guatacondo 4. Ramaditas; 5. Quebrada de Maní 6. Caleta Huelén 42; 7. Loa W3; 8. Loa River; 9. Salado River/El Sifón; 10. Salado River/Las Juntas; 11. Chiu Chiu 200; 12. Confluencia 1; 13. Confluencia 2; 14. Chiu Chiu Cementerio; 15. Calar 1; 16. Talabre Viejo; 17. Tarajne; 18. Vegas de Tilocalar; 19. Finca Chañaral).

*Prosopis* species present in the Atacama Desert belong to the Strombocarpa or the Algarobia section. These can be found at up to 3000 masl of elevation throughout the western Andean slope, and are typically confined to discrete zones of groundwater discharge and/or on riverbanks of perennial/ephemeral watersheds from the Pampa del Tamarugal basin to the Salar the Atacama [45–48] (Fig 2A and 2B). Dominant stands in the Pampa del Tamarugal forest are mostly Strombocarpa, including two endemic species *Prosopis tamarugo* and *Prosopis burkartii*, as well as *Prosopis strombulifera*, which is common in northern Chile and northwestern Argentina [29, 49–51].

Species of the Algarobia section are more widely distributed and range from the Pampa del Tamarugal to the Salar de Atacama basin. There is no consensus as to which species of this section currently grows in the Atacama, although most have been attributed to the Chilenses series (Table 1), [29, 45, 47, 52].

**Table 1:** Atacama Desert Algarobia section species.

Series	Species Algarobia section	Synonymns
Chilenses	*P*. *alba* Gris.	*P*. *atacamensis* Phil.
Chilenses	*P*. *alba* var. *Panta* Gris.	——-
Chilenses	*P*. *flexuosa* DC.	*P*. *fruticose* Meyen
Chilenses	*P*. *chilensis* Mol.	*P*. *siliquastrum* DC.
Chilenses	*P*. *nigra* Gris.	——-

The biogeography of the Algarobia section is complex. Roig [34] suggested a radiation centre located in the Chaco ecoregion, and subsequent migrations to the southern and western arid regions of South America. As with other legumes common to arid zones, Algarobia could have migrated across the tropics by successfully colonizing one patch after another [35]. According to Bessega et al. [38] and Catalano et al. [32] the diversification and dispersal of the Chilenses series in the Chaco lowlands was closely linked to the onset of aridity in the late Pliocene/Pleistocene, but the timing of dispersion remains controversial. A Pliocene/Pleistocene age implies that Algarobia species expanded into the Atacama Desert after the uplift of the Andes [33, 53, 54], which represents a prominent biogeographical barrier for east-west biotic exchanges in southern South America for at least the past 13 million years.

A rich South American megafauna of frugivores was well suited for long-distance dispersal of seeds [55], and could have facilitated the dispersal of Algarrobo [56]. Though, most of these animals became extinct during the late Pleistocene in the Atacama Desert [57–59], several millennia before Algarrobos appeared there (see results). Endozoocharic dispersal has also been documented for smaller mammals, [29, 60–62] but these would result in radiation rates that are to slow to account for observed genetic distances and disjunct distributions among the *Prosopis* species from the Algarobia section [38, 63]. Even though several native species such as guanacos and foxes have been reported as seed dispersers [64] their effect is often on a local scale and such mammal vectors are not effective over long distances [65]. There are no records of *Prosopis* being dispersed by birds [38], or these vectors are otherwise considered minor seed disseminators [33].

Under such a scenario, how and when did the Chilenses series *Prosopis* species cross over the Andes into the hyperarid Atacama Desert? What dispersal mechanisms were involved? Or perhaps the puzzling biogeography of the Algarobia section points to a human pre-Columbian dispersal event or events?

## Material and methods

To establish the arrival and spatial distribution of Algarrobo in the Atacama Desert, we analysed bibliographical references and different geohistorical records from the northern (18–21°S), central (21–24°S) and southern (24–27°S) Atacama Desert (S1 Text).

We also examined in detail the reported presence of Algarobia remains in 17 available pre-Formative and Early Formative archaeological sites from museum collections, as well from data available in archaeological databases (S1 Table). 207 rodent middens (nest deposits built of feces, plant materials, insects, vertebrate remains and encased in urine) collected from 16 different sites nearby archaeological settlements [see 66, 67 for data collection] were also included in our dataset (S2 Table). Finally, we analysed naturally occurring leaf-litter deposits [68] from the southern Pampa del Tamarugal basin that have yielded Algarrobo macrofossils (S2 Table) (i.e. leaves, stems, pods).

All available and purportedly older (than late Holocene) archaeological and palaeoecological remains positively identified as Algarrobo were directly AMS-radiocarbon dated (see Fig 2C). For archaeological samples, specimens found in contexts earlier than ca. 3700 yr BP were given priority for AMS dating. Radiocarbon ages reported here are calibrated using CALIB 7.0.4 at 2-sigma using the Southern Hemisphere calibration curve (SHCal13 Intercept Method); [69] and are given in calendar years before 1950 (cal yr BP).

## Results

**Fig 3:**
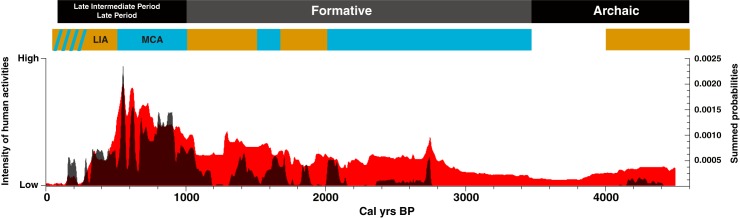
The intensity of human activities in the Atacama Desert (red curve, from [70]) compared to a summed probability distribution of all AMS radiocarbon dates on *Prosopis* Algarobia remains from archaeological and paleoecological contexts (dark curve). The top bar indicates the major cultural periods and paleoclimate variations. Blue bars (orange) represent past wet (dry) climate anomalies. MCA: Medieval Climate Anomaly. LIA: Little ice Age.

The majority of our direct AMS dates on Algarrobo are from the last 3000 cal yr BP (Fig 3).

A total of 18 specimens from 11 archaeological sites were found and sampled, with a mean of 1036 cal yr BP (S3 Table). The archaeological literature shows scant macroscopic evidence for the presence of Algarrobo before the Formative period [18–23, 71, 72] whereas it became very abundant afterwards [10–15] (see S4 Table). Some preceramic sites (we could not obtain access either Tulan or Cañamo), exhibit an even younger taxon specific ^14^C chronology, with a mean of 581 cal yr BP. This demonstrates the importance of direct dating when tracking the introduction of specific archaeological features. Preliminary microfossil analyses on processing instruments also indicate that *Prosopis* species are absent before the onset of the Formative [20]. From a total of 207 scanned rodent middens, only 13 contained Algarrobo endocarps. These were all directly dated and gave a mean age of 970 cal yr BP (S3 Table). Previous plant macrofossils from rodent middens, show that Algarrobo appeared as recently as 700 cal yr BP in the Rio Salado, a tributary of the Loa River [e.g. 66]. Pollen records from rodent middens collected in the Loa basin and oases of the Salar de Atacama (2000–2400 masl) show that *Prosopis* pollen appears after 400 cal yr BP (Maldonado personal communication 2013).

Late Pleistocene (14600–14400 cal yr BP) leaf litter deposits at Lomas de Sal reveal a dense sub-fossil tree community of the endemic *Prosopis tamarugo* (Strombocarpa section) as well as other hygrophytic and phreatophytic trees (*Schinus molle*, *Escallonia angustifolia*, *Myrica pavonis*, etc.) [24, 68, 73]. In contrast, leaves and stems retrieved from late Holocene leaf-litter beds slightly further north (at Quebrada Maní) are Algarobia species (cf. *Prosopis alba*) that date to as early as ~2000 cal yr BP [24, 73] with a mean within of 892 yr cal. BP (S3 Table).

**Fig 4:**
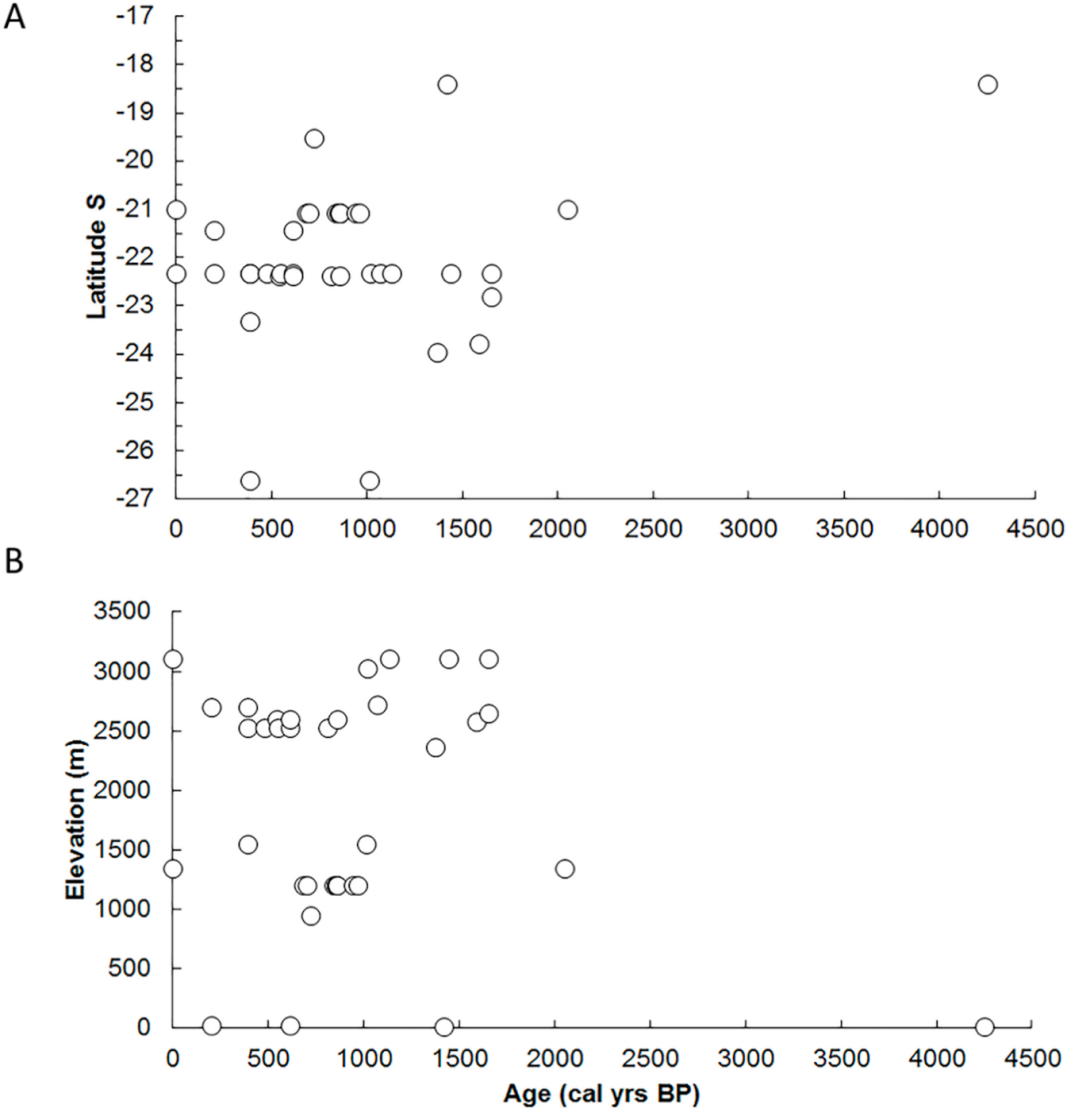
**(A)** A taxon-specific AMS date distribution of the occurrence of Algarrobo remains versus latitude. (**B)** A taxon-specific AMS date distribution of the occurrence of Algarrobo remains versus elevation.

The earliest record of Algarrobo in our sample is a single charred cf. *Prosopis* endocarp which appears at 4250 cal yr BP at our northernmost site along the coast (Lluta 13). Some 2200 years later (at 2050 cal yr BP), Algarrobo appears more than 3° further south at Ramaditas (an archaeological site in Pampa del Tamarugal basin). Algarrobo remains then appear several hundred years later at the higher elevations and increased latitudes of the central Atacama Desert (22–24°S). The earliest presence in the Calama basin is at 1650 cal yr BP (Río Salado/Las Juntas- a rodent midden site) and appears slightly later in the middle Rio Loa and in the Salar de Atacama basin at ca. 1590 cal yr BP. The youngest ages for Algarrobo appearance in our dataset are from the southernmost sites in the Atacama at ca. 1000 cal yr BP (Finca de Chañaral). The remainder of the samples dated all fall within last 1440 cal yr BP or are modern (Fig 4A and 4B).

## Discussion

Literature reviews, macrofossil analyses from palaeoecological and archaeobotanical archives combined with direct dating of Algarobia remains, provide elements for understanding and revaluate the long-term history of Algarrobo in ecosystems of the Atacama over the past 15000 cal yr BP (S3 Table). The Archaic age (4250 cal yr BP) from Lluta 13 in the northernmost sector of the Atacama is an interesting outlier (Fig 4A and 4B) within the site and for the region. One possibility is that it corresponds to *Prosopis pallida* from the Peruvian coast, rather than the nowadays common species in the Atacama (*P*. *flexuosa*, *P*. *alba*). *Prosopis pallida* is part of the Pallidae Series of the Algarobia section and though its origin is intriguing, its distribution is clearly in Peru [33]. In fact, such *Prosopis* remains are ubiquitous in archaeological contexts since the early Holocene along the northern and central coast of Peru [74–77]. Given the paucity of our remains (in contrast to abundant *Prosopis* remains in all our archaeological contexts), only further analyses will confirm if this specimen is part of a wider chronological distribution in the area, or if it is part of the Atacama species (and not from Peru e.g. *P*. *pallida*). Currently there is no information on early archaeobotanical remains in the Argentinian or Bolivian Chaco, although for the northwestern Argentinean Puna, Algarrobo pods are known since 10000 yr BP [78].

The fact that such a multipurpose and invasive species such as Algarrobo does not appear profusely in either the archaeological or palaeoecological records in the Atacama until late in the Holocene has been explained either by seasonality [19, 79, 80], taphonomic biases or a possible range contraction of Algarrobo during a proposed mid-Holocene arid phase [66, 81–83]. A late Holocene appearance would be consistent with known biogeographical data, which points to an origin east of the Andes with a late Pliocene/Pleistocene speciation in the Chaco Region. Its appearance in the Atacama was also contemporaneous with multiple and supra-regional cultural exchanges across the Andes during the Formative. “With introductions made before recorded history, known human population movements, changes in land use and the spread of other plants and animals can be used as indicators of possible spread of *Prosopis*” [33].

In the Atacama, the spread of Algarrobo was contemporaneous with the introduction by humans of other crops such as quinoa, maize, manioc, and sweet potatoes from the high Andes and the tropical forest [84, 85] and with intensified camelid caravan traffic [28]. New settlement patterns in the Atacama Desert might be another indicator for the introduction of these trees. For instance, in the Pampa del Tamarugal the most prominent Formative sedentary villages such as Guatacondo, Ramaditas, Pircas, Caserones and Quebrada Maní (3000–1000 yr BP) arose in previously unoccupied locations [14, 16, 24, 86]. This is also true for sites in the Loa and Salar de Atacama basins, such as Chiu Chiu 200 and Tulor 1 (ca. 3000–1400 yr BP), which shifted away from settlements located in ravines during the Archaic, to the more expansive oases from the Formative onwards [10, 12, 87, 88]. In all of these Formative settlements, the presence of Algarrobo in domestic and burial sites as well as in buried forests in the surroundings is pervasive [11, 12, 15, 88–91].

Whether intended or not, the introduction of given plants is hard to examine in pre-Columbian times [92]. Indeed, a combination of both is possible. The intended cultivation of Algarrobo has been acknowledged by Palacios and Brizuela [93] who stated that “the presence and similarities between Algarrobos from different localities in the Americas are a consequence of their cultivation, and this appears to be a case for domestication that, as with other American crops, did not persist with the arrival of European settlers” (translated from Spanish by the authors). Furthermore, there are several examples of prehistoric tree management [56, 94–98]. Regarding Algarrobo in Atacama, the Spanish historian Oviedo and Valdes [99] in his 1535 chronicle states that Algarrobo woodlands from the San Pedro de Atacama basin oasis were the product of long-lasting farming activities developed by native inhabitants; and Palacios and Brizuela [93] highlight the considerable exo-morphological similarities between these trees and *Prosopis alba* specimens found in the Salta District (NW Argentina). This has led several authors to suggest that *Prosopis alba* may have been introduced from Argentina into the Atacama during pre-Hispanic times [51, 100–102].

As for unintended consequences, *Prosopis* trees are highly invasive, so once brought in they can colonize extensive areas even from single introductions [33]. This could have been facilitated by climate, which became more hospitable in the area after 2500 yrs BP (14–18). In South Africa, the spread of *Prosopis* species followed periods of high rainfall [103, 104] possibly due to improved conditions for germination and establishment or perhaps increased seed dispersal events. A long-term study in northern India found that *Prosopis juliflora* is a pioneer species of denuded or abandoned ravines [105]. In the Atacama, Homero Altamirano (a former national parks ranger) has seen how seeds sprout after being carried by flash floods and it is also common to see fox feces full of seeds [106]. The role that these forests could have had for camelid fodder or other silviopastoralist activities has not been addressed for the Atacama, but has been discussed for prehistoric studies from coastal Peru [107]. In North America, various studies document the encroachment of these trees in grassland ecosystems when herbivores are introduced [108], and although such a mechanism could have preceded humans, it would have intensified when they arrived at suitable areas with domestic and wild animals.

The biogeographical, archaeological and palaeoecological data shown here provides evidence that Algarrobo appeared in the Atacama late in the Holocene (ca. 4000 yr BP or later), and that the most likely vectors were humans. The rapid spread within the Atacama Desert was probably the result of a combination of both natural (geo-dispersal, endozoochary) and cultural actions (either intended as management and cultivation, or unintended such as encroachment of trees due to domestic camelids spreading their seeds near settlements). By ca. 1000 yr BP, these trees became a main resource and colonized landscapes across a wide range of sites and in multiple contexts.

Further dating is needed as new remains appear in different contexts. A critical review of the biogeographic, phylogenetic and taxonomic studies together with better phylogeographic data will also increase our understanding of the presence of this genus in Chile and adjacent areas. Moreover, the relevance of these trees in archaeological and ethnographical contexts implies that we need to better establish the nature of human-Algarrobo interactions. Such assessments will provide new knowledge and challenges for upcoming contingences, as well as a comprehensive approach to indigenous silviculture that succeeded for thousands of years.

## Supporting information

S1 Text Provenience of the samples. (DOCX)

S1 Table Archaeological sites reviewed for this research. (XLSX)

S2 Table Paleo rodent-middens and leaf litter samples reviewed in this research (RM: rodent middens; LL: leaf litter). (XLSX)

S3 Table Chronology and geographical settings for *Prosopis* records retrieved from palaeoecological and archeological archives. n/a indicates no available information. (XLSX)

S4 Table Archaeological sites from which Algarrobo has been assumed to exist pre-3700 yr BP dates. Dates are referential to the sites.
(XLSX)
