# Supplementary material for: The pre-Columbian introduction and dispersal of Algarrobo (Prosopis, Section Algarobia) in the Atacama Desert of northern Chile
Source: PLoS One. 2017 Jul 24;12(7):e0181759. doi: 10.1371/journal.pone.0181759 (PMC5524391; doi:10.1371/journal.pone.0181759)
Supplement: S1 Text — (DOCX) [file pone.0181759.s001.docx]

**S1 Text.** Provenience of the samples

Archaeobotanical evidence for the presence of Algarrobo in Archaic and Formative sites was reviewed from museum databases. Collections directly inspected were from Universidad de Chile, Anthropology Laboratory (Santiago, Chile), Universidad de Antofagasta, Anthropology Laboratory (Antofagasta, Chile), Iquique Regional Museum (Iquique, Chile), Museo Nacional de Historia Natural (Santiago, Chile). Databases were reviewed for Instituto de Investigaciones Canchones (Iquique, Chile) and Museo Universidad de Tarapacá, San Miguel de Azapa (Arica, Chile). A total of 18 specimens from 11 archaeological sites were sampled from Universidad de Chile (with permission of the current authority, Dr. Lorena Sanhueza) and Universidad de Antofagasta (with permission of the current authority, Dr. Agustín Llagostera) (all necessary permits were obtained for the described study, which complied with all relevant regulations).

Rodent middens and leaf litters came from existing laboratory collections housed at the Laboratorio de Paleoecología y Paleoambientes (LP^2^, Departamento de Ecología, Pontificia Universidad Católica de Chile). Modern *Phyllotis* (leaf-eared mice) and *Abrocoma* (chinchilla rat) middens containing ubiquitous Algarrobo pods and/or seeds have been observed in areas where the trees were common, so clearly the rodents will eat Algarrobo pods if available. Indeed, we verified in the field that rodents will usually incorporate Algarrobo mesocarps and seeds into their middens but often leave the endocarps intact. This minimizes any taphonomic biases regarding the preservation/incorporation of Algarrobo macrofossils imposed by foraging behaviour.
